# Discovery and characterization of a novel irreversible EGFR mutants selective and potent kinase inhibitor CHMFL-EGFR-26 with a distinct binding mode

**DOI:** 10.18632/oncotarget.15443

**Published:** 2017-02-17

**Authors:** Chen Hu, Aoli Wang, Hong Wu, Ziping Qi, Xixiang Li, Xiao-E Yan, Cheng Chen, Kailin Yu, Fengming Zou, Wenchao Wang, Wei Wang, Jiaxin Wu, Juan Liu, Beilei Wang, Li Wang, Tao Ren, Shanchun Zhang, Cai-Hong Yun, Jing Liu, Qingsong Liu

**Affiliations:** ^1^ High Magnetic Field Laboratory, Chinese Academy of Sciences, Hefei, Anhui 230031, P. R. China; ^2^ University of Science and Technology of China, Hefei, Anhui 230036, P. R. China; ^3^ CHMFL-HCMTC Target Therapy Joint Laboratory, Hefei, Anhui 230031, P. R. China; ^4^ Institute of Systems Biomedicine, Department of Biophysics, Beijing Key Laboratory of Tumor Systems Biology and Center for Molecular and Translational Medicine, School of Basic Medical Sciences, Peking University Health Science Center, Beijing 100191, P.R. China; ^5^ Precision Targeted Therapy Discovery Center, Institute of Technology Innovation, Hefei Institutes of Physical Science, Chinese Academy of Sciences, Hefei, Anhui 230088, P. R. China; ^6^ Hefei Cosource Medicine Technology Co. LTD., Hefei, Anhui 230031, P. R. China

**Keywords:** EGFR, EGFRT790M, NSCLC, kinase inhibitors, drug resistance

## Abstract

EGFR T790M mutation accounts for about 40-55% drug resistance for the first generation EGFR kinase inhibitors in the NSCLC. Starting from ibrutinib, a highly potent irreversible BTK kinase inhibitor, which was also found to be moderately active to EGFR T790M mutant, we discovered a highly potent irreversible EGFR inhibitor CHMFL-EGFR-26, which is selectively potent against EGFR mutants including L858R, del19, and L858R/T790M. It displayed proper selectivity window between the EGFR mutants and the wide-type. CHMFL-EGFR-26 exhibited good selectivity profile among 468 kinases/mutants tested (S score (1)=0.02). In addition, X-ray crystallography revealed a distinct “DFG-in” and “cHelix-out” inactive binding mode between CHMFL-EGFR-26 and EGFR T790M protein. The compound showed highly potent anti-proliferative efficacy against EGFR mutant but not wide-type NSCLC cell lines through effective inhibition of the EGFR mediated signaling pathway, induction of apoptosis and arresting of cell cycle progression. CHMFL-EGFR-26 bore acceptable pharmacokinetic properties and demonstrated dose-dependent tumor growth suppression in the H1975 (EGFR L858R/T790M) and PC-9 (EGFR del19) inoculated xenograft mouse models. Currently CHMFL-EGFR-26 is undergoing extensive pre-clinical evaluation for the clinical trial purpose.

## INTRODUCTION

EGFR is a receptor tyrosine kinase, which upon ligand EGF (epidermal growth factor) binding will result in auto-phosphorylation and subsequently lead to activation of downstream signaling pathways such as RAS/RAF/ERK, PI3K/AKT, and JAK/STAT pathways [1]. Gain-of-function mutations of EGFR such as del19 and L858R have been observed in 10-35% NSCLC patients [2]. The aberrant activation of EGFR leads to activation of downstream signaling pathways associated with tumor growth and progression [3]. The first generation EGFR kinase inhibitors, such as Gefitinib, have shown great clinical efficacy for the EGFR mutant driven NSCLC, but after a period of treatment, most patients will develop EGFR T790M-induced drug resistance [4]. The drug resistance is mainly caused by the higher binding affinity of ATP in T790M EGFR protein compared to inhibitors, and steric hindrance due to the mutation [4]. Fortunately, T790M mutant of EGFR is still sensitive to the second-generation EGFR inhibitors such as Afatinib, which bind EGFR irreversibly through the formation of covalent bond [5–7]. However, the narrow selectivity window between the EGFR wt and T790M mutant led to side effects, which raised a safety concern for application of these drugs [8]. This encouraged the discovery of the third generation inhibitors, such as AZD9291 and CO-1686, which achieved better selectivity between EGFR wt and T790M mutant [9, 10].

Recently, Ibrutinib, an FDA approved irreversible BTK kinase inhibitor for MCL, CLL etc, has been found to be moderately active against EGFR T790M mutant and has been evaluated in the clinic [11, 12]. However, the efficacy of Ibrutinib for suppression of EGFRT790M mediated tumor progression was limited and required combination of other signaling pathway inhibitors such as MEK inhibitor GSK1120212 [11]. In consideration of these limitations, we developed a novel highly potent EGFR inhibitor CHMFL-EGFR-26 which selectively inhibits EGFR oncogenic mutations including L858R, del19 and L858R/T790M. CHMFL-EGFR-26 irreversibly inhibits EGFR kinase via formation a covalent bond with Cys797 residue in a distinct DFG-in-cHelix-out inactive conformation of EGFR and displays good *in vitro* and *in vivo* anti-NSCLC efficacies in the preclinical models.

## RESULTS

### Rational design of EGFR mutant inhibitor CHMFL-EGFR-26

**Figure 1:**
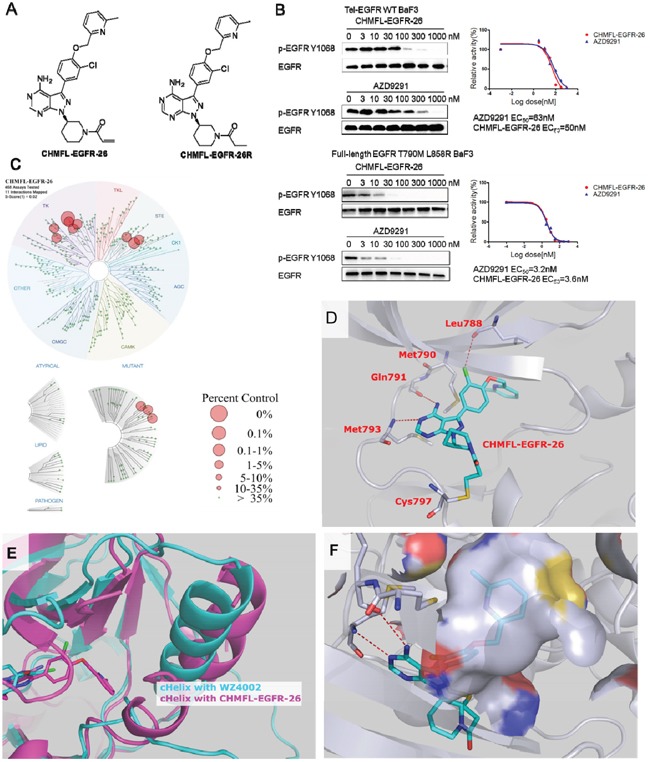
Discovery of CHMFL-EGFR-26 **A**. The chemical structure of CHMFL-EGFR-26 and its reversible version CHMFL-EGFR-26R. **B**. The effects of CHMFL-EGFR-26 and AZD9291 on EGFR Y1068 auto-phosphorylation EGFR wt/L858R/T790M mutants transformed BaF3 isogenic cells. **C**. Treespot demonstration of CHMFL-EGFR-26 selectivity profile against a panel of 468 kinases with DiscoveRx KinomeScan technology at the concentration of 1μM. **D**. X-ray crystal structure of CHMFL-EGFR-26 in complex with EGFR T790M protein (PDB ID: 5GTY). **E**. Superimposition of the EGFR T790M+WZ4002 structure (slate, PDB ID: 3AKI) and the T790M+CHMFL-EGFR-26 structure (pink, PDB ID: 5GTY). **F**. The hydrophobic pocket generated by cHelix-out conformation accommodate the methyl pyridine moiety of CHMFL-EGFR-26.

In our previous research, we have found that the FDA approved irreversible BTK kinase inhibitor Ibrutinib could also selectively and potently inhibit EGFR primary mutants such as L858R and del19 [11]. In addition, it exhibited moderately inhibitory activity against EGFR gatekeeper mutant T790M. Based on the structure of Ibrutinib, we rationally designed a novel inhibitor CHMFL-EGFR-26 which was expected to improve the binding efficiency against EGFR T790M drug resistant mutant meanwhile keep the selectivity over EGFR wt. (Figure 1A, chemical synthetic procedures were listed in the supplemental materials and synthetic scheme was shown in Supplementary Figure 1.) We first tested the anti-proliferative effects of CHMFL-EGFR-26 in a panel of EGFR kinase wt/mutants expressing BaF3 isogenic cell lines (Table 1). The results demonstrated that CHMFL-EGFR-26 potently inhibited EGFR L858R, del19, T790M and L858R/T790M mutants (GI_50_s range from 0.0003 μM to 0.013μM) meanwhile kept over 500-fold selectivity over EGFR wt (GI_50_: 5.4 μM). In addition, it did not exhibit any apparent inhibitory activity against parental BaF3 cells (GI_50_: >10 μM) indicating no general toxicity. For all of the sensitive mutants, when C797S mutant was introduced, CHMFL-EGFR-26 lost activity significantly (50-10000 folds) which suggested an irreversible binding mode via cysteine 797 residue. Furthermore, the reversible version of CHMFL-EGFR-26, which was generated by saturation of acrylamide to propionamide (CHMFL-EGFR-26R, chemical structure shown in Figure 1A), almost completely lost the activity to the CHMFL-EGFR-26 sensitive mutants. This again indicated that CHMFL-EGFR-26 inhibited those EGFR mutants through an irreversible binding mode via cysteine 797 residue. The FDA approved third generation EGFR inhibitor AZD9291 (the chemical structure is showed in Supplementary Figure 3) displayed a similar trend in this growth inhibitory assay except that it also exhibited moderate inhibitory activity against parental BaF3 cells (GI_50_: 1.5 μM versus >10 μM) and the selectivity window between the EGFR mutants and WT was narrower than CHMFL-EGFR-26. The enzymatic inhibition result of CHMFL-EGFR-26 was detected by SelectScreen techonology (Life Technologies). CHMFL-EGFR-26 showed an IC_50_ of 19nM against EGFR T790M mutant, 71nM against EGFR WT and 215 nM against EGFR L858R mutant (Table 2). The selectivity window in biochemical assay was narrower than in the cellular assay between EGFR wt and T790M mutant, we reasoned that this might be due to the different conformations of EGFR kinases *in vitro* and in cell.

**Table 1:** Anti-proliferation effect against CHMFL-EGFR-26 against a panel of BaF3 isogenic cell lines^a^

Cells (GI50: μM)	CHMFL-EGFR-26	CHMFL-EGFR-26R	AZD9291
Parental BaF3	>10	>10	1.5
BaF3-TEL-EGFR	5.4	>10	0.3
BaF3-TEL-EGFR-C797S	2.5	6.6	0.59
BaF3-TEL-EGFR-L858R	<0.0003	>10	<0.0003
BaF3-TEL-EGFR-L858R-C797S	3.2	>10	0.59
BaF3-TEL-EGFR-T790M	<0.0003	>10	0.013
BaF3-TEL-EGFR-T790M-C797S	3.7	>10	0.55
BaF3-FL-EGFR-del19	0.013	>10	0.001
BaF3-FL-EGFR-del19-T790M	<0.0003	7.1	<0.0003
BaF3-FL-EGFR-del19-T790M-C797S	2.1	7.3	0.2
BaF3-FL-EGFR-L858R-T790M	0.006	>10	<0.0003
BaF3-FL-EGFR-L858R-T790M-C797S	0.34	>10	0.31

^a^All GI_50_ values were obtained by triplet testing.

**Table 2:** CHMFL-EGFR-26 biochemical data against EGFR wt/ mutants

Kinases	IC50(nM)
EGFR(ErbB1)	71.4±7.5
EGFR(ErbB1)T790M	19.01±1.62
EGFR(ErbB1)L858R	215.1±2.1

In order to further confirm this compound's on-target effect, we then examined its effect on the blockage of EGFR auto-phosphorylation in EGFR transformed BaF3 isogenic cell lines. The results demonstrated that upon 4h's CHMFL-EGFR-26 treatment, the phosphorylation of EGFR Y1068 was inhibited with an EC_50_ of 3.6 nM in BaF3-FL-EGFR-L858R -T790M cells and 50 nM in BaF3-TEL-EGFR cells (Figure 1B), suggesting that the drug shows 15-fold selectivity between the EGFR wt and EGFRL858R/T790M. Meanwhile, AZD9291 exhibited similar selectivity between the EGFR wt and EGFR L858R/T790M mutant Y1068 auto-phosphorylation (EC_50_: 63nM and 3.2 nM respectively).

### CHMFL-EGFR-26 is a mutant-selective, irreversible inhibitor for EGFR

In order to better understand CHMFL-EGFR-26's selectivity, we then tested it in DiscoveRx's KinomeScan™ assay. The results revealed that it was highly selective among 468 kinases/mutants (S score (1)=0.02) tested at the concentration of 1μM. (Figure 1C and Supplementary Table 1) Besides EGFR wt and mutants, it also displayed strong binding to BLK, BTK, Her2, Her4, JAK3, LOK, MEK1 and MEK5. Given the fact that KinomeScan™ is a binding assay and sometimes cannot truly reflect the compound inhibitory activity, we then used Invitrogen SelectScreen® assay to test these potential off-targets (Supplementary Table 2). The results showed that CHMFL-EGFR-26 also exhibited strong inhibition for BLK (IC_50_=3.9nM), Her4 (IC_50_=5.39nM), Her2 (IC_50_=15.9nM), BTK (IC_50_=16.3nM), BMX (IC_50_=38.9nM), JAK3 (IC_50_=30.6nM) and moderate activity against MEK1 (IC_50_=131nM). This was not surprising because EGFR, JAK3, HER2, HER4 and TEC family kinases (BTK, BLK and BMX) all share a similar cysteine residue at the identical position analogous to Cys 797 in EGFR in the hinge binding area [13]. In addition, considering that some of the EGFR inhibitors induced hyperglycemia in the clinic, which was possibly through inhibition of the INSR and IGF1R kinases, we also tested this drug in parallel with AZD9291 in the INSR and IGF1R transformed isogenic BaF3 cells. (Supplementary Table 3) The results revealed that it was much less potent than AZD9291 against both INSR (GI_50_: 2.8μM versus 0.5 μM) and IGF1R (GI_50_: 3.6 μM versus 0.56 μM) indicating that there was much less chance that this drug could induce the hyperglycemia adverse effects.

To better understand CHMFL-EGFR-26's binding mode, we then tried to obtain its X-ray crystal structure in complex with EGFR T790M mutant (PDB ID: 5GTY, data shown in Supplementary Table 4). As expected, the crystal structure showed that the acrylamide of CHMFL-EGFR-26 formed a covalent bond with Cys797 of EGFR (Figure 1D). The aminopyrimidine formed two hydrogen bonds with Met793 and Gln791 in the hinge binding area. The Met790 oriented in parallel with the cholorophenol moiety and formed a beneficial hydrophobic interaction. Furthermore, the Cl-atom formed a halogen bond with Leu788 carbonyl which presumably contributed to the high binding affinity [14]. Furthermore, it is interesting to note that EGFR T790M adopted a distinct DFG-in-cHelix-out inactive conformation when bound to CHMFL-EGFR-26, which is different from the typical type I EGFR inhibitors such as WZ4002 (PDB ID 3AKI). (Figure 1E) This special conformation generated larger hydrophobic pocket which accommodated the methylpyridine moiety. (Figure 1F).

### CHMFL-EGFR-26 selectively inhibited proliferation of EGFR mutant cell lines

We next tested CHMFL-EGFR-26 on a panel of EGFR wt and mutants NSCLC cell lines. (Table 3) The result demonstrated that CHMFL-EGFR-26 was highly potent against EGFR L858R/T790M mutant expressing cell line H1975 (GI_50_: 0.0003 μM), which was about 170-fold more potent than AZD9291 (GI_50_: 0.052μM). (Table 3) In addition, it also potently inhibited the growth of EGFR del19 expressing cells (PC9 and HCC827: GI_50_: 0.035μM and 0.036 μM) and EGFR L858R expressing cell (H3255 GI_50_: 0.027 μM). It significantly lost activity against EGFR wt expressing NSCLC cell lines such as A549( GI_50_: 5.4μM), H1355 (GI_50_: 8.1 μM), H2122 (GI_50_: 3.5 μM ) and H1703 (GI_50_: 7.7 μM), which is in the similar trend as AZD9291. The washout experiment in H1975, PC9 and H3255 cell lines revealed a time- and dose-dependent blockage of EGFR Y1068 phosphorylation. (Supplementary Figure 2) Combining the results that CHMFL-EGFR-26R, which lost its irreversible binding capability, completely lost activity against those sensitive cell lines, these further confirmed that CHMFL-EGFR-26's irreversible binding mode observed in the X-ray crystal structure was biologically relevant. Furthermore, CHMFL-EGFR-26 did not exhibit any apparent growth inhibitory activity against normal Chinese hamster ovary cells CHO and CHL (GI_50_: >10 μM), indicating a non-general toxicity property.

**Table 3:** CHMFL-EGFR-26 anti-proliferation effect against EGFR wt/mutant NSCLC cell lines^a^

Cell lines (GI50: μM)	CHMFL-EGFR-26	CHMFL-EGFR-26R	AZD9291
H1975 (EGFRL858R/T790M)	<0.0003	>10	0.052
PC-9 (EGFR del19)	0.035	>10	0.002
HCC827 (EGFR del19)	0.036	>10	0.001
H3255 (EGFRL858R)	0.027	>10	0.033
A549 (EGFR wt)	5.4	>10	3.5
H2122 (EGFR wt)	3.5	>10	1.2
H1355(EGFR wt)	8.1	>10	3.0
H1703 (EGFR wt)	7.7	9.4	3.5
CHO	>10	>10	4.2
CHL	>10	>10	2.9

^a^All GI_50_ values were obtained by triplet testing.

### CHMFL-EGFR-26 inhibited EGFR mediated signaling pathways in NSCLC cell lines

**Figure 2:**
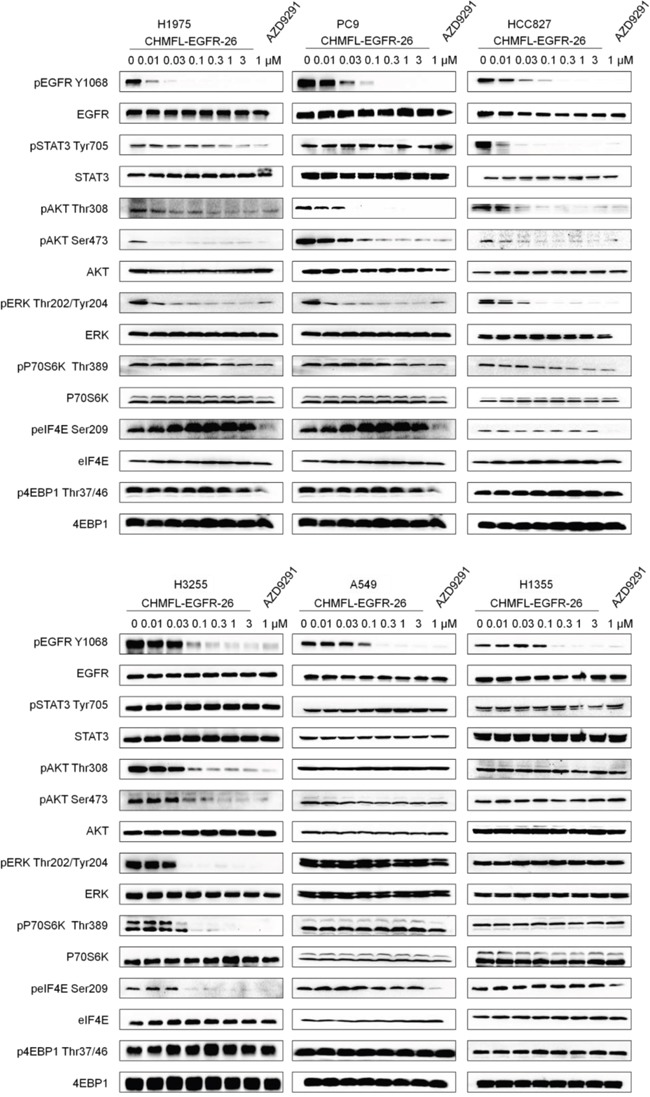
Effect of CHMFL-EGFR-26 on EGFR mediated signal pathway in NSCLC mutants and wt cell lines

We next examined the effects of CHMFL-EGFR-26 on EGFR mediated signaling pathways in NSCLC cell lines with different EGFR mutants. (Figure 2) In the H1975 cell line, 10 nM concentration of the drug could almost completely suppress the autophosphorylation of EGFR Y1068. The downstream phosphorylations of AKT on Thr308, Ser473 and ERK were also inhibited. Similarly, in the PC-9, HCC827 and H3255 cell lines, phosphorylation of EGFR, AKT and ERK were also dose-dependently inhibited, which showed the same trend as AZD9291. As expected, in the EGFR wt expressing NCSCL cell lines A549 and H1355, only autophosphorylation of EGFR was affected and none of the downstream mediators was inhibited. Interestingly, in HCC827 cells, phosphorylation of STAT3 was significantly inhibited (EC_50_: < 10 nM) but this phenomenon was not observed for any other NSCLC cell lines except that in H1975 cells it was weakly blocked starting from the concentration of 1μM. In addition, in H3255 cells, the downstream mediator of mTOR-p70S6K and eIF4E phosphorylations were completely blocked at the concentration of 30 nM, which was not observed in other cell line either. However, in all of the cell lines tested, AZD9291 significantly affected the phosphorylation of eIF4E, which presumably was due to its strong inhibitory activity against MNK2 kinase (IC_50_: 95 nM) [9].

### CHMFL-EGFR-26 induced cell cycle arrest and apoptosis in NSCLC cell lines

**Figure 3:**
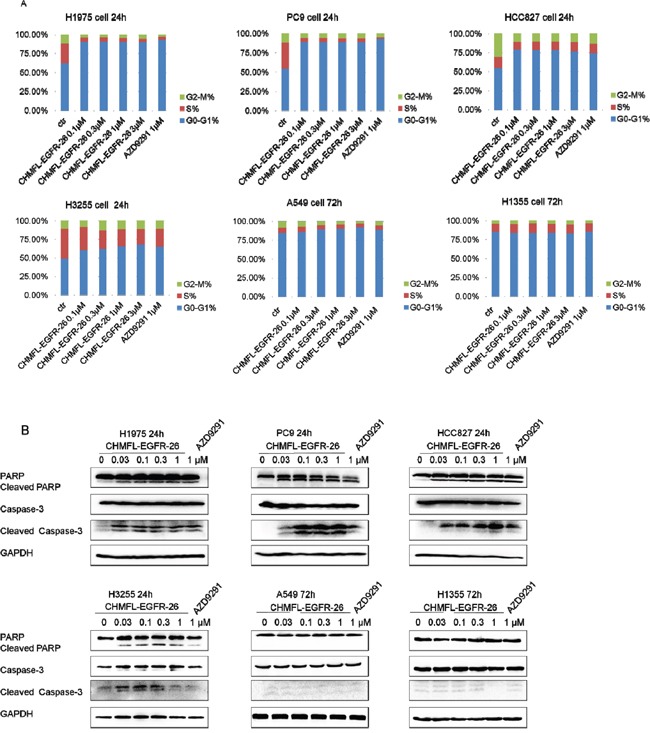
Effect of CHMFL-EGFR-26 on cell cycle progression and apoptosis in EGFR mutants/wt NSCLC cell lines

We then examined CHMFL-EGFR-26's effect on cell cycle progression and apoptosis. Not surprisingly, in the drug sensitive cell lines including H1975, HCC827, PC9 and H3255 which expressed EGFR mutants, CHMFL-EGFR-26 dramatically blocked cell cycle at G0/G1 phase in a dose-dependent manner at 24-hour. While for NSCLC cell lines with wild-type EGFR (A549 and H1355), CHMFL-EGFR-26 did not cause cell cycle arrest at concentrations up to 3 μM after 72-hour treatment. (Figure 3A) CHMFL-EGFR-26 also induced apoptosis in EGFR mutant NSCLC cell lines in a dose-dependent manner (Figure 3B). In H1975, HCC827, PC9 and H3255, 30nM concentration of CHMFL-EGFR-26 was sufficient to induce Caspase-3 and PARP cleavage after 24hrs of treatment. However, in EGFR wt NSCLC cell lines, even after 72hrs drug treatment, there was no cleaved Caspase-3 and PARP detected. This result was correlated with the growth inhibition effects observed in different NSCLC cell lines.

### CHMFL-EGFR-26 suppressed the tumor growth of H1975 and PC9 xenograft model

To further investigate the effect of CHMFL-EGFR-26 *in vivo*, we first examined its pharmacokinetic properties in rats (Supplementary Table 5). The half-life is 0.8 hour by oral dosing which is short but considering the irreversible binding mode, this short half-life was preferred [13]. The compound shows good bioavailability of 33.81%. These results showed that the compound is suitable for oral administration.

**Figure 4:**
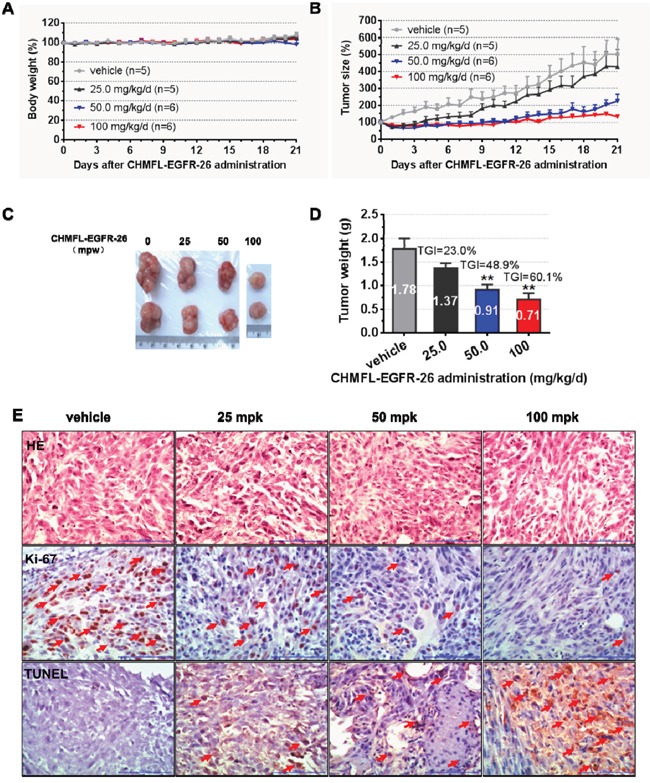
Anti-tumor efficacy of CHMFL-EGFR-26 in H1975 cell inoculated xenograft mouse mode Female nu/nu mice bearing established H1975 tumor xenografts were treated with CHMFL-EGFR-26 at 25.0, 50.0 and 100 mg/kg/d, or vehicle. Daily oral administration was initiated when H1975 tumors had reached a size of 200 to 400 mm^3^. Each group contained 5 or 6 animals. Data, mean ± SEM. **A**. Body weight and **B**. Tumor size measurements from H1975 xenograft mice after CHMFL-EGFR-26 administration. Initial body weight and tumor size were set as 100%. **C**. Representative photographs of tumors in each group after 25.0, 50.0 or 100 mg/kg/d CHMFL-EGFR-26 or vehicle treatment. **D**. Comparison of the final tumor weight in each group after 21-day treatment period. Numbers in columns indicate the mean tumor weight in each group. **p<0.01. **E**. Representative micrographs of hematoxylin and eosin (HE), Ki-67, and TUNEL staining of tumor tissues with CHMFL-EGFR-26 treatment compared to the vehicle-treated group. Note the specific nuclear staining of cells with morphology consistent with proliferation and apoptosis (E, red arrow).

**Figure 5:**
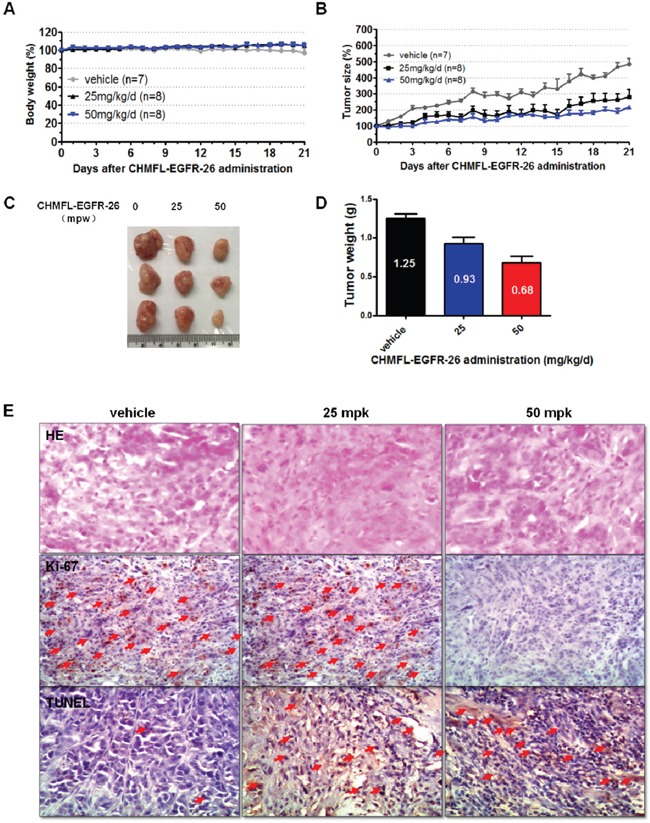
Anti-tumor efficacy of CHMFL-EGFR-26 in PC9 cell inoculated xenograft mouse mode Female nu/nu mice bearing established PC9 tumor xenografts were treated with CHMFL-EGFR-26 at 25.0 and 50.0 mg/kg/d, or vehicle. Daily oral administration was initiated when PC9 tumors had reached a size of 200 to 400 mm^3^. Each group contained 7 or 8 animals. Data, mean ± SEM. **A**. Body weight and **B**. Tumor size measurements from PC9 xenograft mice after CHMFL-EGFR-26 administration. Initial body weight and tumor size were set as 100%. **C**. Representative photographs of tumors in each group after 25.0 or 50.0 mg/kg/d CHMFL-EGFR-26 or vehicle treatment. **D**. Comparison of the final tumor weight in each group after 21-day treatment period. Numbers in columns indicate the mean tumor weight in each group. **E**. Representative micrographs of hematoxylin and eosin (HE), Ki-67, and TUNEL staining of tumor tissues with CHMFL-EGFR-26 treatment compared to the vehicle-treated group. Note the specific nuclear staining of cells with morphology consistent with proliferation and apoptosis (E, red arrow).

In H1975 cells inoculated xenograft mouse model, oral administration of CHMFL-EGFR-26 with different dosages (25, 50 and 100mg/kg/day) did not show any apparent toxicity. (Figure 4A) It also exhibited dose-dependent tumor growth suppression and 100 mg/kg/day dosage could almost completely blocked the tumor progression and exhibited a TGI (tumor growth inhibition rate) of 60.1% (Figure 4B, 4C, 4D). Immunohistochemical (IHC) staining showed that CHMFL-EGFR-26 inhibited the cell proliferation by Ki67 stain and induced apoptosis by TUNEL stain in the tumor tissues. (Figure 4E) Similar results were observed in PC-9 cells inoculated xenograft mouse model. (Figure 5)

## DISCUSSION

As the major drug resistant mechanism for the first line EGFR kinase inhibitors, EGFR T790M mutatioin has attracted extensive attention from the drug discovery community [4, 15]. Since the seminal discovery of the so-called third generation EGFR inhibitors WZ4002 [16], a number of irreversible inhibitors, such as AZD9291, CO-1686, HM61713 (The chemical structures are showed in Supplementary Figure 3), have been developed [9, 10, 17]. However, most of the current EGFR inhibitors bear a similar aminopyridine pharmacophore and the chemical scaffold diversity is highly limited [18]. Given the fact that different pharmacophores provide different drug profiles, ie. drug like property and safety etc, inhibitors based on new scaffolds are always useful and supplementary to the current drug library. CHMFL-EGFR-26, bearing a novel class of pyrazolopyrimidine pharmacophore, was developed from the BTK kinase inhibitor, Ibrutinib, which also exhibited moderate EGFR T790M mutant activity in the preclinical models, by trimming off the BTK kinase activity and improving the EGFR kinase activity, which added one more example of new drug discovery using “old drug repurposing approach” [11, 12].

In the clinical trials of CO-1686 and AZD9291, hyperglycemia has been observed as one of the adverse events, possibly due to the activity against INSR and IGF1R kinases either through themselves or the corresponding metabolites [19–21]. CHMFL-EGFR-26 itself has no apparent activity against these two kinases and in the preclinical mouse model study no hyperglycemia phenomenon was observed, which indicated that it might bear a better therapeutic window for the hyperglycemia. Furthermore, despite of the similar covalent binding via cysteine 797 residues in the EGFR kinase with all other irreversible EGFR kinase inhibitors, CHMFL-EGFR-26 forced the EGFR kinase to adopt a distinct “DFG-in-cHelix-out” inactive conformation, which is rarely observed for the EGFR kinase inhibitors [16, 22]. This might provide a new opportunity for developing the novel inhibition mechanism based EGFR inhibitors and help to reveal new enzymatic biology of EGFR kinase.

It has been reported that in the EGFR mutant driven NSCLCs, STAT3 pathway was activated [23]. Interestingly, we only observed significant inhibition of the phosphorylation of STAT3 in HCC827 cells (EGFR del19) but not any other cell lines, even in PC9 cells, which shares the same EGFR del19 mutant. It has been reported that upon 24hrs of Erlotinib treatment in NSCLC cells, phosphorylation of STAT3 level increased in all the cell lines that express activating EGFR mutants, except HCC827 which lacks the STAT3 feedback loop, suggesting the genetic background of HCC827 may be different in terms of STAT3 signal pathway regulation [24]. Considering that CHMFL-EGFR-26 has no apparent inhibitory activity against SRC and JAK1/2, which are the upstream mediators of STAT3, there must be some other factors contributing to this inhibition and are worth further study [25].

In addition, among all of the EGFR mutants and wt NSCLC cell lines tested, CHMFL-EGFR-26 only inhibited the phosphorylations of eIF4E and p70S6K in H3255 cell line which expressed EGFR L858R mutant. Due to its potent inhibitory activity to the MNK2, which is the upstream mediator of eIF4E, AZD9291 inhibited the phosphorylation of eIF4E in all of the cell lines tested. However, CHMFL-EGFR-26 did not exhibit MNK2/mTOR kinase inhibition in the kinase selectivity profiling [9], which might it might be due to the different genetic background or some other non-kinase based targets that requires further investigation. In addition, it has been reported that in H1975 cells with high mTOR expression, Gefitinib did not suppress mTOR activity, but in H3255 which is low mTOR expressed, Gefitinib treatment inhibited the phosphorylation of mTOR and p70S6K which correlated with the phenomena observed in this work [26].

Furthermore, compared to the clinically used AZD9291, CHMFL-EGFR-26 requires relatively larger dosage to achieve the anti-tumor efficacy. One of the possible reasons is that CHMFL-EGFR-26 bears a relatively shorter half-life (T1/2: 0.8h) compared to AZD9291 (T1/2: 3.1 h). Although the shorter half-life of irreversible inhibitors have been appreciated considering the possible adverse effect during the circulation *in vivo* [13], it may also attenuate the drug's efficacy if the exposure time is not enough and some of the drugs have not been able to form the covalent bond with the target protein yet. Therefore, further medicinal chemistry effort to improve the balance of half-life/residence time associated side effects will be required.

In summary, from the clinically used BTK kinase inhibitor, Ibrutinib's core pharmaophore, we have discovered a novel class of EGFR mutants active irreversible inhibitor CHMFL-EGFR-26. It displayed distinct binding mode, proper drug like properties, potentially better safety window as well as potent *in vitro* and *in vivo* efficacies against EGFR mutants driven NSCLC preclinical models, which makes it a potential useful clinical candidate.

## MATERIALS AND METHODS

### Inhibitors

AZD9291 was purchased from Haoyuan Chemexpress Inc; CHMFL-EGFR-26 and CHMFL-EGFR-26R were synthesized in the lab, and the synthesis procedure is described in the supplementary materials.

### Cell lines and cell culture

The human cancer cell lines H1975, HCC827, H2122, CHO, CHL and H1355 were purchased from the American Type Culture Collection (ATCC) (Manassas, VA, USA). PC9 cell line was purchased from the Sigma-Aldrich (St. Louis, MO, USA). A549, H3255 were purchased from Cobioer Biosciences CO., LTD (Nanjing, China). H1975, PC-9, HCC827 and EGFR mutant isogenic BaF3 cells were cultured in RPMI 1640 media (Corning, USA) with 10% fetal bovine serum (FBS) and supplemented with 2% L-glutamine and 1% penicillin/streptomycin. A549 and H1355 were cultured in F-12K Nutrient Mixture (kaighn's Modification) (Gibco, USA) with 10% FBS and supplemented with 2% L-glutamine and 1% pen/strep. H3255 was cultured in BEGM media (LONZA, USA) with 10% FBS and supplemented with 2% L-glutamine and 1% pen/strep.

We have authenticated the following cell lines through cell line short tandem repeat (STR) profiling (GENEWIZ, Suzhou, China): H1975, PC9, H3255, HCC827, A549, H2122, H1703. All cell lines matched >90% with lines listed in the ATCC, DSMZ or JCRB Cell Line Bank STR Profile Information.

### Antibodies and immunoblotting

The following antibodies were purchased from Cell Signaling Technology (Danvers, MA): EGF Receptor (D38B1) XP® Rabbit mAb(#4267), Phospho-EGF Receptor (Tyr1068) (D7A5) XP® Rabbit mAb (#3777), Stat3 (#9132), Phospho-Stat3 (Tyr705) (D3A7) XP® Rabbit mAb (#9145), Akt (pan) (C67E7) Rabbit mAb(#4691), Phospho-Akt (Ser473) (D9E) XP® Rabbit mAb (#4060), Phospho-Akt (Thr308) (D25E6) XP® Rabbit mAb (#13038), p44/42 MAPK (Erk1/2) (137F5) Rabbit mAb (#4695), Phospho-p44/42 MAPK (Erk1/2) (Thr202/Tyr204) (D13.14.4E) XP® Rabbit mAb(#4370), GAPDH (D16H11) XP® Rabbit mAb, 4E-BP1 (53H11) Rabbit mAb (#9644), eIF4E (C46H6) Rabbit mAb(#2067), Phospho-eIF4E (Ser209) (#9741), PARP Rabbit mAb (#9542), Caspase 3 Rabbit mAb (#9662). Antibodies were used at 1:1000.

### BaF3 isogenic cell line generation

Retroviral constructs for BaF3-TEL-EGFR and BaF3-EGFR variants were made based on the pMSCVpuro (Clontech) backbone. For TEL-fusion vectors, the first 1 kb of human TEL gene with an artificial myristoylation sequence (MGCGCSSHPEDD) was cloned into pMSCVpuro retroviral vector, followed by a 3xFLAG tag sequence and a stop codon. Then the kinase domain coding sequences of EGFR variants were inserted in-frame between TEL and 3xFLAG sequences. For full-length expression vectors, the coding sequences of EGFR variants were directly cloned in pMSCVpuro vector with a 3xFLAG tag at the C-terminal end. All mutations were performed using the QuikChange Site-Directed Mutagenesis Kit (Stratagene) following the manufacturer's instructions. Retrovirus was made using the same method described above and was used to infect BaF3 cells. After puromycin selection, the IL-3 concentration in the medium was gradually withdrawn until cells were able to grow in the absence of IL-3.

### Cell proliferation assays

Cells were grown in 96-well culture plates (2500-3000/well). For adherent cell lines, compounds of various concentrations were added into the plates after cells were cultured for 12 hours. Cell proliferation was determined after treatment with compounds for 72 hours. Cell viability was measured using the CellTiter–Glo assay (Promega, USA) according to the manufacturer's instructions, and luminescence was measured in a multi-label reader (Envision, PerkinElmer, USA). Data were normalized to control groups (DMSO) and represented by the mean of three independent measurements with standard error <20%. GI_50_ values were calculated using Prism 5.0 (GraphPad Software, San Diego, CA).

### Cell cycle analysis

H1975, PC9, HCC827 and H3255 cells were treated with DMSO, CHMFL-EGFR-26 (0.1μM, 0.3μM, 1μM and 3μM) and AZD9291 (1μM) for 24 hours before cells were harvested by trypsin and washed with cold PBS. A549 and H1355 cells were treated with DMSO, CHMFL-EGFR-26 (0.1μM, 0.3μM, 1μM and 3μM) and AZD9291 (1μM) for 72 hours before cells were harvested by trypsin and washed with cold PBS. The cells were fixed in 70% cold ethanol and incubated at −20 °C overnight then stained with PI/RNase staining buffer (BD Pharmingen). Flow cytometry was performed using a FACS Calibur (BD), and results were analyzed by ModFit software.

### Signaling pathway examination

H1975, PC9, HCC827, H3255, A549 and H1355 cells were treated with serially diluted CHMFL-EGFR-26 and AZD9291 (1μM) for 4 hours. Cells were then collected and lysed. Phospho-EGF Receptor, Phospho-EGF Receptor (Tyr1068), Stat3, Phospho-Stat3 (Tyr705), AKT, Phospho-AKT (Ser473), Phospho-AKT (Thr308), p44/42 MAPK (Erk1/2), Phospho-p44/42 MAPK (Erk1/2) (Thr202/ Tyr204), p70 S6 Kinase, Phospho-p70 S6 Kinase (Thr389), eIF4E, Phospho-eIF4E (Ser209), 4E-BP1, Phospho-4E-BP1 (Thr37/46) antibody (Cell Signaling Technology) were used for immunoblotting.

### Washing out experiment

H1975, PC9 and H3255 cells were treated with CHMFL-EGFR-26 (0.01μM, 0.03μM, 0.1μM) for 4 hours before they were washed out by PBS for three times. Then cells were incubated in 10% FBS-containing RPMI for indicated time periods before they were collected and lysed. EGFR, Phospho-EGFR (Tyr1068) antibody (Cell signaling Technology) were used for immunoblotting.

### Apoptosis effect examination

H1975, PC9, HCC827 and H3255 cells were treated with DMSO, CHMFL-EGFR-26 (0.03μM, 0.1μM, 0.3μM and 1μM) and AZD9291 (1μM) for 24 hours before cells were harvested. A549 and H1355 cells were treated with DMSO, CHMFL-EGFR-26 (0.03μM, 0.1μM, 0.3μM and 1μM) and AZD9291 (1μM) for 72 hours. Cells were then washed in PBS and lysed in cell lysis buffer. PARP, Caspase-3, GAPDH antibodies were used for immunoblotting.

### Cloning, Expression, and Purification of EGFR T790M

The EGFR constructs spanning residues 696-1022 harboring the T790M mutation were cloned into the pFastBac HTA vector (Invitrogen). Baculoviruses to express these proteins were then prepared using the Bac-to-Bac Baculovirus expression system (Invitrogen). A 6xHis-tag followed by a linker peptide containing both the Thrombin and Tobacco Etch Virus (TEV) protease cleavage sites was fused to the N-terminal end of the EGFR proteins. The fusion proteins were then expressed in the SF9 insect cells.

Cell pellets were suspended in lysis buffer containing 20mM Tris-HCl (pH8.0), 500mM NaCl, 5mM KCl, 20mM imidazole, 1% Glycerol, 1 mM tris(2-carboxyethyl) phosphine hydrochloride (TCEP), and 1x protease inhibitor mixture (Complete EDTA-free, Roche). The cells were broken by sonication. The lysate was centrifuged at 20,000 rpm for one hour at 4°C, then the supernatant was incubated with Ni-NTA Sepharose beads (GE). The beads were washed with lysis buffer and eluted with the same buffer supplemented with 400mM imidazole. The N-terminal 6xHis-tag plus the linker was removed through incubation with His-tagged TEV protease for 4 hours at 4 °C. The uncleaved protein and the His-tagged TEV protease were removed by passing the mixture through Ni-NTA Sepharose beads and the proteins without the tag were further purified by size-exclusion chromatography (Superdex 200) in the wash buffer. The pure EGFR proteins were concentrated to 15 mg/ml, flash frozen in liquid nitrogen and stored at −80°C for later using.

### Crystallization and structure determination

The EGFR 696-1022 T790M/CHMFL-EGFR-26 complex crystals were both prepared by co-crystallization at 20°C using the handing drop vapor diffusion method. The EGFR proteins were incubated with 1mM compound on ice for 4 hours before setting-up the crystallization trays. The reservoir solution for EGFR 696-1022 T790M CHMFL-EGFR-26 co-crystallization was 0.05M CaCl_2_, 0.1M B-Tris pH 6.2, 24% PEG 550MME, 5mM TCEP. For data collection, all crystals were rapidly dipped in their corresponding reservoir solutions supplemented with 25% ethylene glycol and flash frozen in liquid nitrogen.

X-ray diffraction data were collected at 100K at beamline BL19U1 at Shanghai Synchrotron Radiation Facility (SSRF). The diffraction data were processed using HKL3000 [27]. The structure was determined by molecular replacement with Phaser [28] utilizing the EGFR T790M structure (PDB ID 2JIT)[29] as the search model. Repeated rounds of manual refitting and crystallographic refinement were done using COOT [30] and Phenix [30, 31]. The inhibitor was modeled into the closely fitting positive Fo-Fc electron density and included in following refinement cycles. Topology and parameter files for the inhibitor were obtained using PRODRG [32]. The EGFR 696-1022 T790M/ CHMFL-EGFR-26 complex crystal structure has been deposited in Protein Data Bank with the accession code 5GTY, respectively. The data collection and refinement statistics of the structure are summarized in Supplementary Table 4.

### H1975 and PC9 xenograft tumor model

Five-week old female nu/nu mice were purchased from the Shanghai Experimental Center, Chinese Science Academy (Shanghai, China). All animals were housed in a specific pathogen-free facility and used according to the animal care regulations of Hefei Institutes of Physical Science Chinese Academy of Sciences. Prior to implantation, cells were harvested during exponential growth. Five million cells in PBS were formulated as a 1:1 mixture with Matrigel (BD Biosciences) and injected into the subcutaneous space on the right flank of nu/nu mice. Daily oral administration was initiated when tumors had reached a size of 200 to 400 mm^3^. Animals were then randomized into treatment groups of 5 or 6 mice each for efficacy studies. CHMFL-EGFR-26 was delivered daily in a HKI solution (0.5% Methocellulose/0.4% Tween80 in ddH_2_O) by orally gavage. A range of doses of CHMFL-EGFR-26 or its vehicle were administered, as indicated in figure legends. Body weight and tumor growth was measured daily after CHMFL-EGFR-26 treatment. Tumor volumes were calculated as follows: tumor volume (mm^3^)= [(W^2^ × L)/2] in which width (W) is defined as the smaller of the two measurements and length (L) is defined as the larger of the two measurements.

### Immunohistochemistry stain

Tumor tissues were fixed in 10% neutral-buffered formalin and embedded in paraffin. Six-micron tissue section were prepared, deparaffinized, dehydrated, and then stained with hematoxylin and eosin (H&E) using routine methods. Commercially available primary antibody to human Ki-67 (ZSGB-BIO, Beijing, China) was used for Ki-67 staining. After heat-induced antigen retrieval, formalin-fixed and paraffin-embedded tumor tissue sections were stained with primary antibody overnight at 4°C. The slides were subsequently incubated with ImmPRES anti-mouse Ig (Vector Laboratories, Burlingame, CA) at room temperature for 30 min, stained with peroxidase substrate 3,3’-diaminobenzidine chromogen (Vector Laboratories), and finally counterstained with hematoxylin. TUNEL staining was assessed using *In Situ* Cell Death Detection Kit (POD) (Roche, Mannheim, Germany) according to the manufacturer's instructions.

## SUPPLEMENTARY MATERIALS FIGURES AND TABLES





## References

[1] Prenzel N, Fischer OM, Streit S, Hart S, Ullrich A (2001). The epidermal growth factor receptor family as a central element for cellular signal transduction and diversification. Endocr Relat Cancer.

[2] Pao W, Chmielecki J (2010). Rational, biologically based treatment of EGFR-mutant non-small-cell lung cancer. Nat Rev Cancer.

[3] Dearden S, Stevens J, Wu YL, Blowers D (2013). Mutation incidence and coincidence in non small-cell lung cancer: meta-analyses by ethnicity and histology (mutMap). Ann Oncol.

[4] Yun CH, Mengwasser KE, Toms AV, Woo MS, Greulich H, Wong KK, Meyerson M, Eck MJ (2008). The T790M mutation in EGFR kinase causes drug resistance by increasing the affinity for ATP. Proc Natl Acad Sci U S A.

[5] Li D, Ambrogio L, Shimamura T, Kubo S, Takahashi M, Chirieac LR, Padera RF, Shapiro GI, Baum A, Himmelsbach F, Rettig WJ, Meyerson M, Solca F, Greulich H, Wong KK (2008). BIBW2992, an irreversible EGFR/HER2 inhibitor highly effective in preclinical lung cancer models. Oncogene.

[6] Ou SH (2012). Second-generation irreversible epidermal growth factor receptor (EGFR) tyrosine kinase inhibitors (TKIs): a better mousetrap? A review of the clinical evidence. Crit Rev Oncol Hematol.

[7] Smaill JB, Rewcastle GW, Loo JA, Greis KD, Chan OH, Reyner EL, Lipka E, Showalter HD, Vincent PW, Elliott WL, Denny WA (2000). Tyrosine kinase inhibitors. 17. Irreversible inhibitors of the epidermal growth factor receptor: 4-(phenylamino)quinazoline- and 4-(phenylamino)pyrido[3,2-d]pyrimidine-6-acrylamides bearing additional solubilizing functions. J Med Chem.

[8] Yap TA, Popat S (2014). Toward precision medicine with next-generation EGFR inhibitors in non-small-cell lung cancer. Pharmgenomics Pers Med.

[9] Cross DA, Ashton SE, Ghiorghiu S, Eberlein C, Nebhan CA, Spitzler PJ, Orme JP, Finlay MR, Ward RA, Mellor MJ, Hughes G, Rahi A, Jacobs VN (2014). AZD9291, an irreversible EGFR TKI, overcomes T790M-mediated resistance to EGFR inhibitors in lung cancer. Cancer Discov.

[10] Walter AO, Sjin RT, Haringsma HJ, Ohashi K, Sun J, Lee K, Dubrovskiy A, Labenski M, Zhu Z, Wang Z, Sheets M, T St Martin, Karp R (2013). Discovery of a mutant-selective covalent inhibitor of EGFR that overcomes T790M-mediated resistance in NSCLC. Cancer Discov.

[11] Wu H, Wang A, Zhang W, Wang B, Chen C, Wang W, Hu C, Ye Z, Zhao Z, Wang L, Li X, Yu K, Liu J (2015). Ibrutinib selectively and irreversibly targets EGFR (L858R, Del19) mutant but is moderately resistant to EGFR (T790M) mutant NSCLC Cells. Oncotarget.

[12] Gao W, Wang M, Wang L, Lu H, Wu S, Dai B, Ou Z, Zhang L, Heymach JV, Gold KA, Minna J, Roth JA, Hofstetter WL, Swisher SG, Fang B (2014). Selective antitumor activity of ibrutinib in EGFR-mutant non-small cell lung cancer cells. J Natl Cancer Inst.

[13] Li X, Zuo Y, Tang G, Wang Y, Zhou Y, Wang X, Guo T, Xia M, Ding N, Pan Z (2014). Discovery of a series of 2,5-diaminopyrimidine covalent irreversible inhibitors of Bruton's tyrosine kinase with in vivo antitumor activity. J Med Chem.

[14] Ford MC, Ho PS (2016). Computational Tools To Model Halogen Bonds in Medicinal Chemistry. J Med Chem.

[15] Lynch TJ, Bell DW, Sordella R, Gurubhagavatula S, Okimoto RA, Brannigan BW, Harris PL, Haserlat SM, Supko JG, Haluska FG, Louis DN, Christiani DC, Settleman J, Haber DA (2004). Activating mutations in the epidermal growth factor receptor underlying responsiveness of non-small-cell lung cancer to gefitinib. N Engl J Med.

[16] Zhou W, Ercan D, Chen L, Yun CH, Li D, Capelletti M, Cortot AB, Chirieac L, Iacob RE, Padera R, Engen JR, Wong KK, Eck MJ, Gray NS, Janne PA (2009). Novel mutant-selective EGFR kinase inhibitors against EGFR T790M. Nature.

[17] Wang S, Cang S, Liu D (2016). Third-generation inhibitors targeting EGFR T790M mutation in advanced non-small cell lung cancer. J Hematol Oncol.

[18] Yun CH, Boggon TJ, Li Y, Woo MS, Greulich H, Meyerson M, Eck MJ (2007). Structures of lung cancer-derived EGFR mutants and inhibitor complexes: mechanism of activation and insights into differential inhibitor sensitivity. Cancer Cell.

[19] Goldman JW, Mendenhall MA, Rettinger SR (2016). Hyperglycemia Associated With Targeted Oncologic Treatment: Mechanisms and Management. Oncologist.

[20] Villadolid J, Ersek JL, Fong MK, Sirianno L, Story ES (2015). Management of hyperglycemia from epidermal growth factor receptor (EGFR) tyrosine kinase inhibitors (TKIs) targeting T790M-mediated resistance. Transl Lung Cancer Res.

[21] Andrew D, Simmons SJ-T, Haringsma Henry J., Allen Andrew, Harding. Thomas C (2015). Abstract 793: Insulin-like growth factor 1 (IGF1R)/insulin receptor (INSR) inhibitory activity of rociletinib (CO-1686) and its metabolites in nonclinical models. Cancer Research.

[22] Wang A, Yan XE, Wu H, Wang W, Hu C, Chen C, Zhao Z, Zhao P, Li X, Wang L, Wang B, Ye Z, Wang J (2016). Ibrutinib targets mutant-EGFR kinase with a distinct binding conformation. Oncotarget.

[23] Haura EB, Zheng Z, Song L, Cantor A, Bepler G (2005). Activated epidermal growth factor receptor-Stat-3 signaling promotes tumor survival in vivo in non-small cell lung cancer. Clin Cancer Res.

[24] Lee HJ, Zhuang G, Cao Y, Du P, Kim HJ, Settleman J (2014). Drug resistance via feedback activation of Stat3 in oncogene-addicted cancer cells. Cancer Cell.

[25] Darnell JE, Kerr IM, Stark GR (1994). Jak-STAT pathways and transcriptional activation in response to IFNs and other extracellular signaling proteins. Science.

[26] Karachaliou N, Codony-Servat J, Teixido C, Pilotto S, Drozdowskyj A, Codony-Servat C, Gimenez-Capitan A, Molina-Vila MA, Bertran-Alamillo J, Gervais R, Massuti B, Moran T, Majem M (2015). BIM and mTOR expression levels predict outcome to erlotinib in EGFR-mutant non-small-cell lung cancer. Sci Rep.

[27] Minor W, Cymborowski M, Otwinowski Z, Chruszcz M (2006). HKL-3000: the integration of data reduction and structure solution--from diffraction images to an initial model in minutes. Acta crystallographica Section D, Biological crystallography.

[28] McCoy AJ, Grosse-Kunstleve RW, Adams PD, Winn MD, Storoni LC, Read RJ (2007). Phaser crystallographic software. Journal of applied crystallography.

[29] Zhang X, Gureasko J, Shen K, Cole PA, Kuriyan J (2006). An allosteric mechanism for activation of the kinase domain of epidermal growth factor receptor. Cell.

[30] Emsley P, Cowtan K (2004). Coot: model-building tools for molecular graphics. Acta Crystallogr D Biol Crystallogr.

[31] Adams PD, Afonine PV, Bunkoczi G, Chen VB, Davis IW, Echols N, Headd JJ, Hung LW, Kapral GJ, Grosse-Kunstleve RW, McCoy AJ, Moriarty NW, Oeffner R (2010). PHENIX: a comprehensive Python-based system for macromolecular structure solution. Acta Crystallogr D Biol Crystallogr.

[32] Schuttelkopf AW, van Aalten DM (2004). PRODRG: a tool for high-throughput crystallography of protein-ligand complexes. Acta Crystallogr D Biol Crystallogr.

