# Supplementary material for: Discovery and characterization of a novel irreversible EGFR mutants selective and potent kinase inhibitor CHMFL-EGFR-26 with a distinct binding mode
Source: Oncotarget. 2017 Feb 17;8(11):18359–72. doi: 10.18632/oncotarget.15443 (PMC5392334; doi:10.18632/oncotarget.15443)
Supplement: Supplementary file 1 [file oncotarget-08-18359-s001.pdf]

Discovery and characterization of a novel irreversible EGFR mutants selective and potent kinase inhibitor CHMFL-EGFR-26 with a distinct binding mode

SUPPLEMENTARY DATA

SUPPLEMENTARY MATERIALS AND METHODS

Synthesis procedure

All solvents and reagents were used as obtained. ^{1}H NMR spectra and ^{13}C NMR spectra were recorded with a Bruker 400 NMR spectrometer and referenced to deuterium dimethyl sulfoxide (DMSO-d_6) or deuterium chloroform ($CDCl_3$). Chemical shifts are expressed in ppm. In the NMR tabulation, s indicates singlet; d, doublet; t, triplet; q, quartet; m, multiplet; and br, broad peak. LC/MS experiments were performed on an Agilent 6224 TOF using an ESI source coupled to an Agilent 1260 Infinity HPLC system operating in reverse mode with an Agilent Eclipse Plus C18 1.8 µm, 3.0 mm × 50 mm column. Purification of final compounds were performed with Isco CombiFlash Rf + system (silica flash column 4 g) using a gradient of 0−10% MeOH in DCM over 20 min at a flow rate of 18 mL/min. The purities of all compounds were above 95% (Supplementary Figure 1)[1].

2-((4-bromo-2-chlorophenoxy)methyl)-6-methylpyridine (S1) The mixture of 4-bromo-2-chlorophenol (2.0 g, 9.7 mmol), 2-(bromomethyl)-6-methylpyridine (1.8 g, 9.7 mmol) and K_2CO_3 (1.3 g, 9.7 mmol) in DMF (10 mL) was stirred at room temperature for 2 h. The resulting mixture was diluted with 100 mL of EtOAc, then washed with water (3 × 50 mL) followed by brine. The organic layers were dried over sodium sulfate, filtered, and concentrated to provide **S1** as an a light yellow solid (2.0 g, 66.7%) without further purification. ^{1}H NMR (400 MHz, DMSO-d_6) δ 7.80 – 7.62 (m, 2H), 7.47 (d, J = 5.9 Hz, 1H), 7.33 (s, 1H), 7.18 (d, J = 8.1 Hz, 2H), 5.23 (s, 2H), 2.48 (s, 3H); ^{13}C NMR (100 MHz, DMSO-d_6) δ 158.02, 155.70, 153.48, 137.75, 132.46, 131.48, 123.33, 122.84, 118.69, 116.39, 112.63, 71.67, 24.37; TOF LC/MS: (ESI) m/z: 312.9861 $[M+H]^+$.

2-((2-chloro-4-(4,4,5,5-tetramethyl-1,3,2-dioxaborolan-2-yl)phenoxy)methyl)-6-methylpyridine ***(S2)*** To a solution of ***S1*** (1.5 g, 4.8 mmol) in 1,4-dioxane (15 mL) were added bis(pinacolato)diboron (1.3 g, 5.3 mmol), KOAc (1.0 g, 9.6 mmol), and Pd(dppf)Cl_2 (0.19 g, 0.24 mmol) at room temperature. The reaction mixture was stirred overnight at 100 °C under argon protection. The resulting mixture was concentrated and purified by silica gel column chromatography (eluting with 0−2% MeOH in DCM) to afford **S2** as a light yellow solid (1.1 g, 64.7%). ^{1}H NMR (400 MHz, DMSO-d_6) δ 7.73 (s, 1H), 7.65 (s, 1H), 7.58 (s, 1H), 7.33 (s, 1H), 7.21 (s, 2H), 5.27 (s, 2H), 2.49 (s, 3H), 1.25 (d, J = 22.0 Hz, 12H); ^{13}C NMR (100 MHz, DMSO-d_6) δ 158.02, 156.37, 155.77, 137.75, 136.12, 135.28, 122.81, 121.82, 118.65, 116.34, 114.23, 84.25, 71.33, 25.07, 24.36; LC/MS (ESI, m/z): 360.1532 $[M+H]^+$.

(R)-3-(3-chloro-4-((6-methylpyridin-2-yl)methoxy)phenyl)-1-(piperidin-3-yl)-1H-pyrazolo[3,4-d]pyrimidin-4-amine ***(S4)*** To a mixture of ***S2*** (0.8 g, 2.2 mmol) and 1,4-dioxane/H_2O (25 mL, v/v, 5/1) was added **S3** (1.0 g, 2.3 mmol REF: 1), K_2CO_3 (0.6 g, 4.4 mmol), and $Pd(PPh_3)_4$ (0.1 g, 0.1 mmol). The reaction mixture was placed into an oil bath preheated to 90 °C, with stirring at this temperature for 12 h under argon. The resulting mixture was then concentrated to afford the crude product, which was purified by silica gel column chromatography (eluting with 0−2% MeOH in DCM) to provide the desired Boc-protected monoamine as an off-white solid (0.9 g). Then to the solid in EtOAc (3 mL) was added 4 N HCl in EtOAc (12 mL). The reaction mixture was stirred at room temperature for 1 h. After complete conversion of the starting material, excess EtOAc was removed under vacuum. The residue was diluted in EtOAc and water. The water layer was basified with 2 N $NaHCO_3$ solution and extracted with EtOAc (3 × 70 mL). The organic layers were then washed with water followed by brine. The organic layers were dried over sodium sulfate, filtered, and concentrated to provide **S4** as an off-white solid (0.39 g, 60.0%) without further purification. ^{1}H NMR (400 MHz, DMSO-d_6) δ 8.24 (s, 1H), 7.78 (s, 1H), 7.70 (s, 1H), 7.58 (s, 1H), 7.40 (s, 2H), 7.25 (s, 1H), 5.31 (s, 2H), 4.69 (s, 1H), 3.51 (s, 1H), 3.07 (s, 1H), 2.95 (s, 2H), 2.07 (d, J = 33.3 Hz, 2H), 1.77 (s, 1H), 1.59 (s, 1H); ^{13}C NMR (100 MHz, DMSO-d_6) δ 158.68, 158.06, 155.97, 155.92, 154.21, 154.17, 142.33, 137.84, 130.15, 128.74, 122.88, 122.48, 118.75, 115.09, 97.83, 71.64, 54.35, 51.16, 45.82, 30.67, 26.36, 24.40; LC/MS (ESI, m/z): 450.11815 $[M+H]^+$.

General Method A:

(R)-1-(3-(4-amino-3-(3-chloro-4-((6-methylpyridin-2-yl)methoxy)phenyl)-1H-pyrazolo[3,4-d]pyrimidin-1-yl)piperidin-1-yl)prop-2-en-1-one (**CHMFL-EGFR-26**) To a solution of **S4** (30 mg, 0.06 mmol) in dichloromethane (3 mL) were added DIPEA (9.4 mg, 0.07 mmol) and

acryloyl chloride (6.6 mg, 0.07 mmol) at 0 °C. The resulting mixture was stirred for 5 min. Then it was quenched by MeOH (1 mL), concentrated, and purified with CombiFlash system (0−10% MeOH in DCM) to afford the title compound **CHMFL-EGFR-26** (25.0 mg, 75.7%) as a white solid. ^{1}H NMR (400 MHz, DMSO-d_6) δ 8.29 (s, 1H), 7.76 (d, J = 27.5 Hz, 2H), 7.58 (s, 1H), 7.42 (s, 2H), 7.27 (s, 1H), 6.81 (d, J = 65.3 Hz, 1H), 6.12 (s, 1H), 5.67 (d, J = 51.3 Hz, 1H), 5.32 (s, 2H), 4.73 (s, 1H), 4.53 (s, 0.5H), 4.18 (s, 1H), 4.09 (s, 0.5H), 3.74 (s, 0.5H), 3.26 (s, 1H), 3.08 (s, 0.5H), 2.51 (s, 3H), 2.26 (s, 1H), 2.14 (s, 1H), 1.96 (s, 1H), 1.60 (s, 1H); ^{13}C NMR (100 MHz, DMSO-d_6) δ 165.03, 158.71, 158.04, 156.14, 155.91, 154.45, 154.24, 142.81, 137.79, 130.19, 128.73, 127.87, 127.65, 127.11, 122.84, 122.53, 118.71, 115.04, 97.90, 71.65, 53.18, 52.48, 49.72, 46.19, 45.66, 42.10, 30.07, 29.80, 25.35, 24.39, 23.70; LC/MS (ESI, m/z): 504.1920 $[M+H]^+$.

(R)-1-(3-(4-amino-3-(3-chloro-4-((6-methylpyridin-2-yl)methoxy)phenyl)-1H-pyrazolo[3,4-d]pyrimidin-1-yl)piperidin-1-yl)propan-1-one ***(CHMFL-EGFR-26R)***.

^{1}H NMR (400 MHz, DMSO-d_6) δ 8.26 (s, 1H), 7.79 (s, 1H), 7.71 (s, 1H), 7.58 (s, 1H), 7.40 (s, 2H), 7.25 (s, 1H), 5.32 (s, 2H), 4.74 (s, 1H), 4.63 (s, 0.5H), 4.52 (s, 1H), 4.23 (s, 0.5H), 4.02 (s, 1H), 3.61 (s, 1H), 2.39 (s, 2H), 2.25 (s, 1H), 2.11 (s, 1H), 1.89 (s, 1H), 1.62 (s, 1H), 1.01 (s, 3H); LC/MS (ESI, m/z): 506.2075 $[M+H]^+$.

REFERENCE

1. Li X, Wang A, Yu K, Qi Z, Chen C, Wang W, Wu H, Wu J, Zhao Z, Liu J, Zou F, Wang L. Discovery of (R)-1-(3-(4-Amino-3-(4-phenoxyphenyl)- 1H-pyrazolo[3,4-d]pyrimidin-1-yl)piperidin- 1-yl)-2- (dimethylamino)ethanone (CHMFL-FLT3-122) as a Potent and Orally Available FLT3 Kinase Inhibitor for FLT3-ITD Positive Acute Myeloid Leukemia. J Med Chem. 2015; 58:9625-38. doi 10.1021/acs.jmedchem.5b01611.

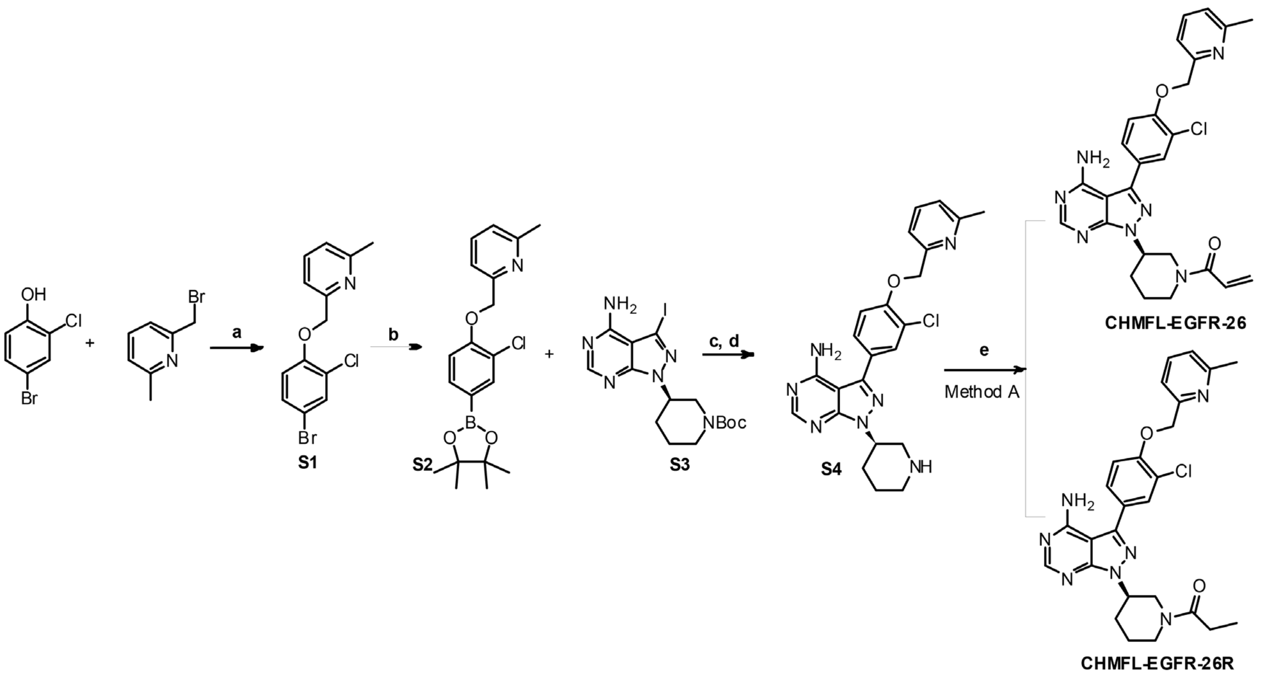

Supplementary Figure 1: Synthesis procedures of CHMFL-EGFR-26 and CHMFL-EGFR-26R. Reagents and conditions: **a.** DMF, K_2CO_3, rt, 2 h; **b.** bis(pinacolato)diboron, 1,4-dioxane, KOAc, Pd(dppf)Cl_2, 100 °C, 12 h; **c.** 1,4-dioxane/H_2O, K_2CO_3, $Pd(PPh_3)_4$, 100 °C, 12 h; **d.** 4 N HCl in EtOAc, rt, 1 h; **e.** acryloyl chloride or propionyl chloride, DIPEA, DCM, 0 °C, 5 min.

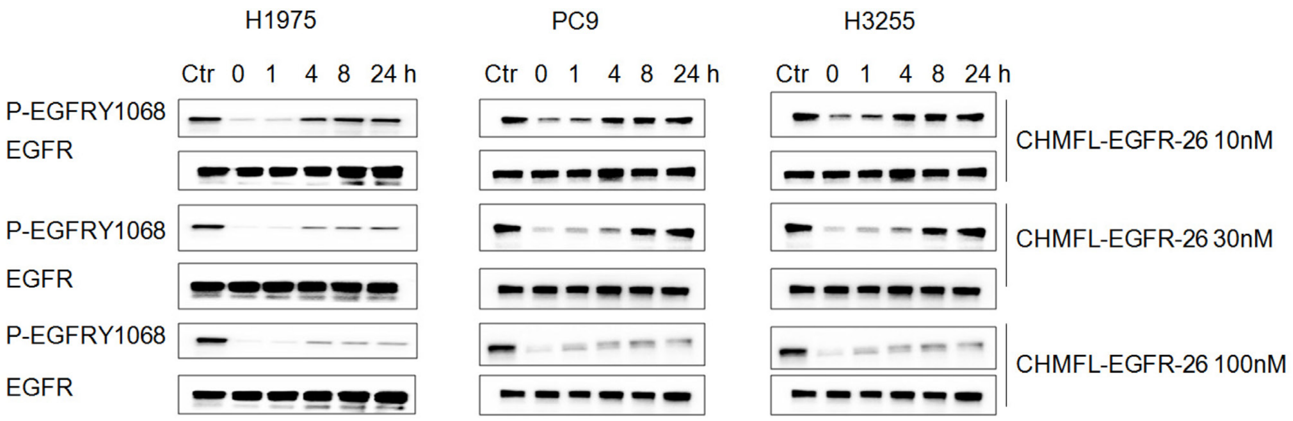

Supplementary Figure 2: Washout experiment in NSCLC mutant cell lines H1975(EGFRL858R/T790M), PC9(EGFR del19) and H3255 (EGFR L858R).

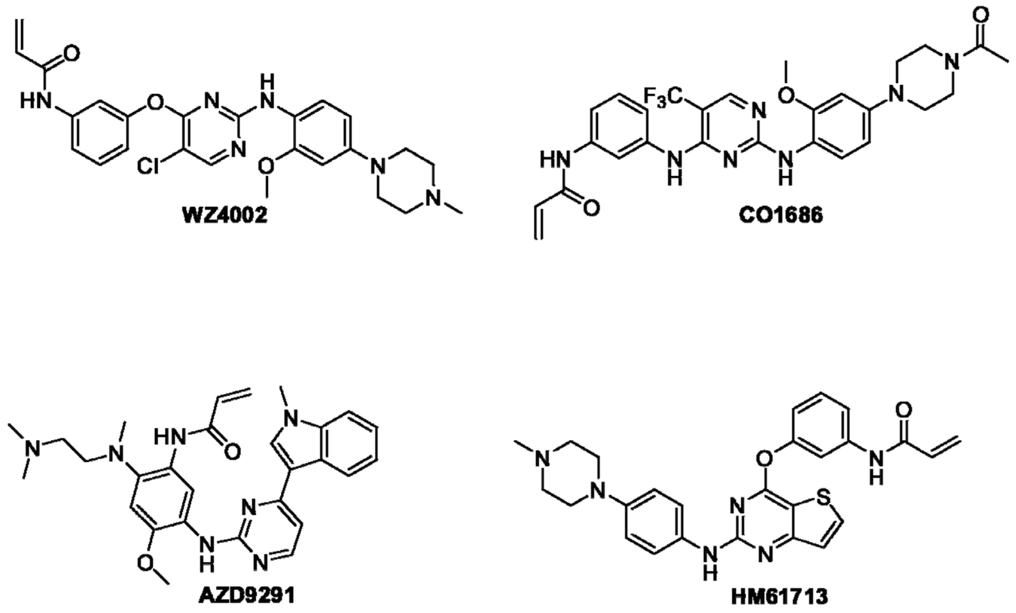

Supplementary Figure 3: The chemical structure of EGFR inhibitors as we mentioned in the discussion section.

Supplementary Table 1: Kinome wide selectivity profiling of CHMFL-EGFR-26 with DiscoveRx's KinomeScan assay

See Supplementary File 1

Supplementary Table 2: biochemical assay of CHMFL-EGFR-26 against kinase targets revealed from the KinomeScan Profiling by invitrogen SelectScreen® assay

	IC50(nM)
BLK	3.9±0.8
BMX	38.9±5.9
BTK	16.3±3.6
HER2	15.9±2.6
HER4	5.39±0.01
JAK3	30.6±4.3
MEK1	131.0±4.3

Supplementary Table 3: CHMFL-EGFR-26 and AZD9291 anti-proliferative effects against INSR and IGF1R transformed isogenic BaF3 cells

IC50(μM)	BaF3-INSR	BaF3-IGF1R	Parental BaF3
AZD9291	0.5	0.56	1.2
CHMFL-EGFR-26	2.8	3.6	9.0

Supplementary Table 4: Data collection and structure refinement statistics of EGFR 696-1022 T790M/ CHMFL-EGFR-26 crystal structure

	EGFR 696-1022 T790M/ CHMFL-EGFR-26
**Data collection**	
Space group	$P2_1$
Cell dimensions	
a, *b*, *c* (Å)	117.4, 71.8, 152.5
α, β, γ (°)	90.0, 103.5, 90.0
Resolution (Å)	50-3.15 (3.26-3.15) *
R_{pim} (%)	11.9 (38.4)
$I/\sigma I$	6.1 (2.1)
Completeness (%)	98.2 (97.0)
Redundancy	5.1 (4.6)
**Refinement**	
Resolution (Å)	49.81-3.14
No. reflections	41550
R_{work} / R_{free}	0.256/0.279
No. atoms	
Protein	18674
Ligand/ion	58
Water	303
B-factors	
Protein	53.5
Ligand/ion	21.2
Water	45.0
R.m.s. deviations	
Bond lengths (Å)	0.010
Bond angles (°)	1.374
Ramachandran plot	
Favored (%)	97.13
Allowed (%)	2.87
Disallowed (%)	0

*Values in parentheses are for highest-resolution shell. One crystal was used to collect this data set.

Supplementary Table 5: Pharmacokinetic parameters of CHMFL-EGFR-26 were calculated on rats

CHMFL-EGFR-26	I.V (1mg/kg)	P.O. (10mg/kg)
AUC(0-t) (ng/mL*h)	1194.35±142.39	4038.57±716.26
AUC(0-∞) (ng/mL*h)	1194.68± 142.58	4039.62±717.16
MRT(0-t) (h)	0.18±0.035	0.72±0.069
$T_{1/2}$ (h)	0.31±0.082	0.74±0.15
T_{max} (h)	0.017±0	0.33±0.14
Vz (L/kg)	0.38±0.13	2.62±0.14
CLz (L/h/kg)	0.85±0.11	2.53±0.45
Cmax (ng/mL)	10645.68±1649.58	5207.02±858.64
F%	/	33.81
