# Supplementary material for: Discovery and characterization of a novel irreversible EGFR mutants selective and potent kinase inhibitor CHMFL-EGFR-26 with a distinct binding mode
Source: Oncotarget. 2017 Feb 17;8(11):18359–72. doi: 10.18632/oncotarget.15443 (PMC5392334; doi:10.18632/oncotarget.15443)
Supplement: Supplementary file 2 [file oncotarget-08-18359-s002.doc]

**Supplemental Table 1**: Kinome wide selectivity profiling of CHMFL-EGFR-26 with DiscoveRx’s KinomeScan assay.

| **DiscoveRx Gene Symbol** | **CHMFL-EGFR-26 (% control)** |
| --- | --- |
| **Concentrations** | **1μM** |
| **AAK1** | 96 |
| **ABL1(E255K)-phosphorylated** | 98 |
| **ABL1(F317I)-nonphosphorylated** | 100 |
| **ABL1(F317I)-phosphorylated** | 98 |
| **ABL1(F317L)-nonphosphorylated** | 100 |
| **ABL1(F317L)-phosphorylated** | 95 |
| **ABL1(H396P)-nonphosphorylated** | 100 |
| **ABL1(H396P)-phosphorylated** | 100 |
| **ABL1(M351T)-phosphorylated** | 100 |
| **ABL1(Q252H)-nonphosphorylated** | 100 |
| **ABL1(Q252H)-phosphorylated** | 100 |
| **ABL1(T315I)-nonphosphorylated** | 100 |
| **ABL1(T315I)-phosphorylated** | 98 |
| **ABL1(Y253F)-phosphorylated** | 100 |
| **ABL1-nonphosphorylated** | 87 |
| **ABL1-phosphorylated** | 81 |
| **ABL2** | 99 |
| **ACVR1** | 96 |
| **ACVR1B** | 30 |
| **ACVR2A** | 89 |
| **ACVR2B** | 85 |
| **ACVRL1** | 100 |
| **ADCK3** | 100 |
| **ADCK4** | 100 |
| **AKT1** | 98 |
| **AKT2** | 86 |
| **AKT3** | 59 |
| **ALK** | 100 |
| **ALK(C1156Y)** | 100 |
| **ALK(L1196M)** | 100 |
| **AMPK-alpha1** | 79 |
| **AMPK-alpha2** | 90 |
| **ANKK1** | 94 |
| **ARK5** | 77 |
| **ASK1** | 97 |
| **ASK2** | 68 |
| **AURKA** | 97 |
| **AURKB** | 77 |
| **AURKC** | 87 |
| **AXL** | 83 |
| **BIKE** | 96 |
| **BLK** | 0.35 |
| **BMPR1A** | 100 |
| **BMPR1B** | 97 |
| **BMPR2** | 100 |
| **BMX** | 27 |
| **BRAF** | 85 |
| **BRAF(V600E)** | 62 |
| **BRK** | 26 |
| **BRSK1** | 93 |
| **BRSK2** | 97 |
| **BTK** | 0.45 |
| **BUB1** | 76 |
| **CAMK1** | 100 |
| **CAMK1B** | 82 |
| **CAMK1D** | 91 |
| **CAMK1G** | 97 |
| **CAMK2A** | 99 |
| **CAMK2B** | 97 |
| **CAMK2D** | 91 |
| **CAMK2G** | 95 |
| **CAMK4** | 100 |
| **CAMKK1** | 88 |
| **CAMKK2** | 75 |
| **CASK** | 95 |
| **CDC2L1** | 86 |
| **CDC2L2** | 97 |
| **CDC2L5** | 100 |
| **CDK11** | 100 |
| **CDK2** | 100 |
| **CDK3** | 84 |
| **CDK4** | 100 |
| **CDK4-cyclinD1** | 100 |
| **CDK4-cyclinD3** | 100 |
| **CDK5** | 100 |
| **CDK7** | 100 |
| **CDK8** | 92 |
| **CDK9** | 90 |
| **CDKL1** | 100 |
| **CDKL2** | 100 |
| **CDKL3** | 97 |
| **CDKL5** | 100 |
| **CHEK1** | 100 |
| **CHEK2** | 88 |
| **CIT** | 90 |
| **CLK1** | 94 |
| **CLK2** | 96 |
| **CLK3** | 100 |
| **CLK4** | 100 |
| **CSF1R** | 98 |
| **CSF1R-autoinhibited** | 100 |
| **CSK** | 32 |
| **CSNK1A1** | 100 |
| **CSNK1A1L** | 91 |
| **CSNK1D** | 95 |
| **CSNK1E** | 9.1 |
| **CSNK1G1** | 87 |
| **CSNK1G2** | 100 |
| **CSNK1G3** | 98 |
| **CSNK2A1** | 100 |
| **CSNK2A2** | 91 |
| **CTK** | 95 |
| **DAPK1** | 77 |
| **DAPK2** | 100 |
| **DAPK3** | 98 |
| **DCAMKL1** | 88 |
| **DCAMKL2** | 95 |
| **DCAMKL3** | 100 |
| **DDR1** | 85 |
| **DDR2** | 90 |
| **DLK** | 78 |
| **DMPK** | 66 |
| **DMPK2** | 28 |
| **DRAK1** | 100 |
| **DRAK2** | 91 |
| **DYRK1A** | 100 |
| **DYRK1B** | 85 |
| **DYRK2** | 86 |
| **EGFR** | 0.8 |
| **EGFR(E746-A750del)** | 34 |
| **EGFR(G719C)** | 16 |
| **EGFR(G719S)** | 18 |
| **EGFR(L747-E749del, A750P)** | 10 |
| **EGFR(L747-S752del, P753S)** | 20 |
| **EGFR(L747-T751del,Sins)** | 21 |
| **EGFR(L858R)** | 2.5 |
| **EGFR(L858R,T790M)** | 2.8 |
| **EGFR(L861Q)** | 0.9 |
| **EGFR(S752-I759del)** | 12 |
| **EGFR(T790M)** | 0.25 |
| **EIF2AK1** | 100 |
| **EPHA1** | 42 |
| **EPHA2** | 100 |
| **EPHA3** | 89 |
| **EPHA4** | 100 |
| **EPHA5** | 100 |
| **EPHA6** | 97 |
| **EPHA7** | 100 |
| **EPHA8** | 89 |
| **EPHB1** | 96 |
| **EPHB2** | 100 |
| **EPHB3** | 100 |
| **EPHB4** | 100 |
| **EPHB6** | 82 |
| **ERBB2** | 0 |
| **ERBB3** | 89 |
| **ERBB4** | 0.45 |
| **ERK1** | 91 |
| **ERK2** | 94 |
| **ERK3** | 100 |
| **ERK4** | 83 |
| **ERK5** | 100 |
| **ERK8** | 91 |
| **ERN1** | 81 |
| **FAK** | 90 |
| **FER** | 56 |
| **FES** | 97 |
| **FGFR1** | 100 |
| **FGFR2** | 100 |
| **FGFR3** | 100 |
| **FGFR3(G697C)** | 100 |
| **FGFR4** | 100 |
| **FGR** | 86 |
| **FLT1** | 93 |
| **FLT3** | 95 |
| **FLT3(D835H)** | 100 |
| **FLT3(D835V)** | 70 |
| **FLT3(D835Y)** | 97 |
| **FLT3(ITD)** | 87 |
| **FLT3(ITD,D835V)** | 99 |
| **FLT3(ITD,F691L)** | 100 |
| **FLT3(K663Q)** | 97 |
| **FLT3(N841I)** | 93 |
| **FLT3(R834Q)** | 100 |
| **FLT3-autoinhibited** | 99 |
| **FLT4** | 99 |
| **FRK** | 96 |
| **FYN** | 90 |
| **GAK** | 72 |
| **GCN2(Kin.Dom.2,S808G)** | 90 |
| **GRK1** | 88 |
| **GRK2** | 99 |
| **GRK3** | 100 |
| **GRK4** | 88 |
| **GRK7** | 100 |
| **GSK3A** | 100 |
| **GSK3B** | 97 |
| **HASPIN** | 100 |
| **HCK** | 44 |
| **HIPK1** | 98 |
| **HIPK2** | 100 |
| **HIPK3** | 100 |
| **HIPK4** | 100 |
| **HPK1** | 100 |
| **HUNK** | 72 |
| **ICK** | 90 |
| **IGF1R** | 100 |
| **IKK-alpha** | 98 |
| **IKK-beta** | 98 |
| **IKK-epsilon** | 100 |
| **INSR** | 100 |
| **INSRR** | 100 |
| **IRAK1** | 100 |
| **IRAK3** | 94 |
| **IRAK4** | 100 |
| **ITK** | 96 |
| **JAK1(JH1domain-catalytic)** | 91 |
| **JAK1(JH2domain-pseudokinase)** | 84 |
| **JAK2(JH1domain-catalytic)** | 95 |
| **JAK3(JH1domain-catalytic)** | 0.7 |
| **JNK1** | 76 |
| **JNK2** | 59 |
| **JNK3** | 86 |
| **KIT** | 100 |
| **KIT(A829P)** | 100 |
| **KIT(D816H)** | 100 |
| **KIT(D816V)** | 100 |
| **KIT(L576P)** | 91 |
| **KIT(V559D)** | 100 |
| **KIT(V559D,T670I)** | 100 |
| **KIT(V559D,V654A)** | 89 |
| **KIT-autoinhibited** | 95 |
| **LATS1** | 82 |
| **LATS2** | 96 |
| **LCK** | 33 |
| **LIMK1** | 99 |
| **LIMK2** | 100 |
| **LKB1** | 79 |
| **LOK** | 0.2 |
| **LRRK2** | 100 |
| **LRRK2(G2019S)** | 100 |
| **LTK** | 94 |
| **LYN** | 94 |
| **LZK** | 86 |
| **MAK** | 100 |
| **MAP3K1** | 82 |
| **MAP3K15** | 100 |
| **MAP3K2** | 52 |
| **MAP3K3** | 67 |
| **MAP3K4** | 86 |
| **MAP4K2** | 100 |
| **MAP4K3** | 97 |
| **MAP4K4** | 46 |
| **MAP4K5** | 54 |
| **MAPKAPK2** | 89 |
| **MAPKAPK5** | 100 |
| **MARK1** | 100 |
| **MARK2** | 95 |
| **MARK3** | 100 |
| **MARK4** | 100 |
| **MAST1** | 93 |
| **MEK1** | 0.3 |
| **MEK2** | 1.1 |
| **MEK3** | 98 |
| **MEK4** | 80 |
| **MEK5** | 0.1 |
| **MEK6** | 95 |
| **MELK** | 41 |
| **MERTK** | 79 |
| **MET** | 96 |
| **MET(M1250T)** | 90 |
| **MET(Y1235D)** | 55 |
| **MINK** | 29 |
| **MKK7** | 2.5 |
| **MKNK1** | 100 |
| **MKNK2** | 91 |
| **MLCK** | 100 |
| **MLK1** | 99 |
| **MLK2** | 80 |
| **MLK3** | 100 |
| **MRCKA** | 64 |
| **MRCKB** | 80 |
| **MST1** | 61 |
| **MST1R** | 100 |
| **MST2** | 53 |
| **MST3** | 39 |
| **MST4** | 99 |
| **MTOR** | 100 |
| **MUSK** | 84 |
| **MYLK** | 100 |
| **MYLK2** | 100 |
| **MYLK4** | 100 |
| **MYO3A** | 97 |
| **MYO3B** | 98 |
| **NDR1** | 71 |
| **NDR2** | 100 |
| **NEK1** | 92 |
| **NEK10** | 99 |
| **NEK11** | 100 |
| **NEK2** | 18 |
| **NEK3** | 83 |
| **NEK4** | 100 |
| **NEK5** | 93 |
| **NEK6** | 100 |
| **NEK7** | 90 |
| **NEK9** | 94 |
| **NIK** | 100 |
| **NIM1** | 96 |
| **NLK** | 70 |
| **OSR1** | 95 |
| **p38-alpha** | 86 |
| **p38-beta** | 100 |
| **p38-delta** | 83 |
| **p38-gamma** | 100 |
| **PAK1** | 59 |
| **PAK2** | 77 |
| **PAK3** | 62 |
| **PAK4** | 94 |
| **PAK6** | 74 |
| **PAK7** | 67 |
| **PCTK1** | 100 |
| **PCTK2** | 89 |
| **PCTK3** | 94 |
| **PDGFRA** | 100 |
| **PDGFRB** | 94 |
| **PDPK1** | 97 |
| **PFCDPK1(P.falciparum)** | 54 |
| **PFPK5(P.falciparum)** | 100 |
| **PFTAIRE2** | 97 |
| **PFTK1** | 100 |
| **PHKG1** | 100 |
| **PHKG2** | 100 |
| **PIK3C2B** | 100 |
| **PIK3C2G** | 100 |
| **PIK3CA** | 100 |
| **PIK3CA(C420R)** | 100 |
| **PIK3CA(E542K)** | 93 |
| **PIK3CA(E545A)** | 100 |
| **PIK3CA(E545K)** | 81 |
| **PIK3CA(H1047L)** | 81 |
| **PIK3CA(H1047Y)** | 100 |
| **PIK3CA(I800L)** | 100 |
| **PIK3CA(M1043I)** | 100 |
| **PIK3CA(Q546K)** | 83 |
| **PIK3CB** | 94 |
| **PIK3CD** | 100 |
| **PIK3CG** | 100 |
| **PIK4CB** | 100 |
| **PIKFYVE** | 75 |
| **PIM1** | 97 |
| **PIM2** | 100 |
| **PIM3** | 100 |
| **PIP5K1A** | 100 |
| **PIP5K1C** | 67 |
| **PIP5K2B** | 100 |
| **PIP5K2C** | 85 |
| **PKAC-alpha** | 100 |
| **PKAC-beta** | 14 |
| **PKMYT1** | 92 |
| **PKN1** | 99 |
| **PKN2** | 53 |
| **PKNB(M.tuberculosis)** | 100 |
| **PLK1** | 100 |
| **PLK2** | 89 |
| **PLK3** | 85 |
| **PLK4** | 99 |
| **PRKCD** | 100 |
| **PRKCE** | 99 |
| **PRKCH** | 70 |
| **PRKCI** | 100 |
| **PRKCQ** | 57 |
| **PRKD1** | 85 |
| **PRKD2** | 81 |
| **PRKD3** | 92 |
| **PRKG1** | 14 |
| **PRKG2** | 100 |
| **PRKR** | 80 |
| **PRKX** | 81 |
| **PRP4** | 78 |
| **PYK2** | 55 |
| **QSK** | 71 |
| **RAF1** | 97 |
| **RET** | 95 |
| **RET(M918T)** | 100 |
| **RET(V804L)** | 100 |
| **RET(V804M)** | 93 |
| **RIOK1** | 90 |
| **RIOK2** | 100 |
| **RIOK3** | 82 |
| **RIPK1** | 100 |
| **RIPK2** | 13 |
| **RIPK4** | 96 |
| **RIPK5** | 13 |
| **ROCK1** | 100 |
| **ROCK2** | 100 |
| **ROS1** | 89 |
| **RPS6KA4(Kin.Dom.1-N-terminal)** | 11 |
| **RPS6KA4(Kin.Dom.2-C-terminal)** | 100 |
| **RPS6KA5(Kin.Dom.1-N-terminal)** | 100 |
| **RPS6KA5(Kin.Dom.2-C-terminal)** | 100 |
| **RSK1(Kin.Dom.1-N-terminal)** | 97 |
| **RSK1(Kin.Dom.2-C-terminal)** | 83 |
| **RSK2(Kin.Dom.1-N-terminal)** | 95 |
| **RSK2(Kin.Dom.2-C-terminal)** | 100 |
| **RSK3(Kin.Dom.1-N-terminal)** | 99 |
| **RSK3(Kin.Dom.2-C-terminal)** | 85 |
| **RSK4(Kin.Dom.1-N-terminal)** | 100 |
| **RSK4(Kin.Dom.2-C-terminal)** | 72 |
| **S6K1** | 100 |
| **SBK1** | 100 |
| **SGK** | 100 |
| **SgK110** | 100 |
| **SGK2** | 100 |
| **SGK3** | 83 |
| **SIK** | 69 |
| **SIK2** | 93 |
| **SLK** | 8.8 |
| **SNARK** | 25 |
| **SNRK** | 100 |
| **SRC** | 100 |
| **SRMS** | 11 |
| **SRPK1** | 100 |
| **SRPK2** | 100 |
| **SRPK3** | 90 |
| **STK16** | 100 |
| **STK33** | 100 |
| **STK35** | 23 |
| **STK36** | 92 |
| **STK39** | 92 |
| **SYK** | 94 |
| **TAK1** | 100 |
| **TAOK1** | 100 |
| **TAOK2** | 78 |
| **TAOK3** | 100 |
| **TBK1** | 100 |
| **TEC** | 13 |
| **TESK1** | 93 |
| **TGFBR1** | 7 |
| **TGFBR2** | 100 |
| **TIE1** | 69 |
| **TIE2** | 78 |
| **TLK1** | 84 |
| **TLK2** | 80 |
| **TNIK** | 11 |
| **TNK1** | 64 |
| **TNK2** | 93 |
| **TNNI3K** | 89 |
| **TRKA** | 55 |
| **TRKB** | 100 |
| **TRKC** | 100 |
| **TRPM6** | 100 |
| **TSSK1B** | 89 |
| **TSSK3** | 100 |
| **TTK** | 95 |
| **TXK** | 15 |
| **TYK2(JH1domain-catalytic)** | 100 |
| **TYK2(JH2domain-pseudokinase)** | 97 |
| **TYRO3** | 53 |
| **ULK1** | 85 |
| **ULK2** | 97 |
| **ULK3** | 92 |
| **VEGFR2** | 100 |
| **VPS34** | 89 |
| **VRK2** | 95 |
| **WEE1** | 85 |
| **WEE2** | 84 |
| **WNK1** | 100 |
| **WNK2** | 92 |
| **WNK3** | 100 |
| **WNK4** | 86 |
| **YANK1** | 3.6 |
| **YANK2** | 5.8 |
| **YANK3** | 90 |
| **YES** | 65 |
| **YSK1** | 20 |
| **YSK4** | 67 |
| **ZAK** | 100 |
| **ZAP70** | 100 |
